# VEGF—Virus Interactions: Pathogenic Mechanisms and Therapeutic Applications

**DOI:** 10.3390/cells13211815

**Published:** 2024-11-04

**Authors:** Cristina Sánchez-Martínez, Esther Grueso, Tania Calvo-López, Jorge Martinez-Ortega, Ana Ruiz, José M. Almendral

**Affiliations:** 1 Biosciences Research Institute, School of Experimental Sciences, Universidad Francisco de Vitoria, Pozuelo de Alarcón, 28223 Madrid, Spain; cristina.sanchez@ufv.es (C.S.-M.); esther.grueso@ufv.es (E.G.); 2 Centro de Biología Molecular Severo Ochoa (CSIC-UAM), Cantoblanco, 28049 Madrid, Spain or tania.calvo@cib.csic.es (T.C.-L.); jorge.martinezortega@uam.es (J.M.-O.); anaruizmedina26@gmail.com (A.R.); 3 Departamento de Biología Molecular, Universidad Autónoma de Madrid, Cantoblanco, 28049 Madrid, Spain; 4 Department of Biomedicine, Centro de Investigaciones Biológicas Margarita Salas (CSIC), Ramiro de Maeztu 9, 28040 Madrid, Spain

**Keywords:** VEGF, angiogenesis, tumor viruses, bevacizumab, oncolytic viruses, VEGF peptides, viral capsids, AAV vectors

## Abstract

Many types of viruses directly or indirectly target the vascular endothelial growth factor (VEGF) system, which is a central regulator of vasculogenesis and angiogenesis in physiological homeostasis, causing diverse pathologies. Other viruses have been developed into effective therapeutic tools for VEGF modulation in conditions such as cancer and eye diseases. Some viruses may alter the levels of VEGF in the pathogenesis of respiratory syndromes, or they may encode VEGF-like factors, promoting vascular disruption and angiogenesis to enable viruses’ systemic spread. Oncogenic viruses may express interactive factors that perturb VEGF’s functional levels or downstream signaling, which increases the neovascularization and metastasis of tumors. Furthermore, many viruses are being developed as therapeutic vectors for vascular pathologies in clinical trials. Major examples are those viral vectors that inhibit the role of VEGF in the neovascularization required for cancer progression; this is achieved through the induction of immune responses, by exposing specific peptides that block signaling or by expressing anti-VEGF and anti-VEGF receptor-neutralizing antibodies. Other viruses have been engineered into effective pro- or anti-angiogenesis multitarget vectors for neovascular eye diseases, paving the way for therapies with improved safety and minimal side effects. This article critically reviews the large body of literature on these issues, highlighting those contributions that describe the molecular mechanisms, thus expanding our understanding of the VEGF–virus interactions in disease and therapy. This could facilitate the clinical use of therapeutic virus vectors in precision medicine for the VEGF system.

## 1. Introduction

As infectious entities with diverse biological properties, many viruses interact with the vasculature under development and/or at mature stages. An understanding of the pathological or therapeutic implications of these interactions requires in-depth knowledge of both the viruses’ life cycles and their genetic organization, as well as those factor networks that regulate the vasculature’s biology, which is briefly discussed here. Vasculogenesis, or the de novo growth of new blood vessels from endothelial stem cells, occurs during embryogenesis [1]. Here, angiogenesis, or the formation of new capillary structures from pre-existing ones [2], may take place during physiological processes (e.g., pregnancy, the menstrual cycle, muscle growth, tissue remodeling, or wound healing) or several pathologies (immune diseases, eye diseases, or ischemic and inflammatory disorders) [3]. Of particular biomedical interest is the angiogenesis associated with tumor growth, a concept introduced by Judah Folkman in 1971 [4], as it increases the provision of O_2_ and nutrients to tumor cells, facilitating their invasion and metastasis [5,6]. Indeed, blocking tumor angiogenesis hinders tumor growth and may even lead to complete remission [7,8,9].

Multiple types of cancer secrete a plethora of angiogenic factors (growth factors, cytokines, proteases, inhibitors, endogenous modulators, and others), which occurs through cancer cells themselves or through cells in the tumor microenvironment (associated fibroblasts, pericytes, and immune cells), to induce vascularization via the activation of the pre-existing host endothelium. Among these factors, the members of the Vascular Endothelial Growth Factor (VEGF) family play a major role in neovascularization. VEGF is a 45 kDa heparin-binding polypeptide mitogen with target specificity for vascular endothelial cells [10,11]. It is widely distributed in most tissue types and organs but particularly in areas of active vascular proliferation, including numerous types of human tumors [12]. As outlined below, the key role of VEGF in tumor angiogenesis was highlighted when it was found that its inhibition prevented tumor growth in vivo [13,14], providing the first evidence that targeting a paracrine mediator of the vasculature significantly inhibits tumor growth. Subsequently, VEGF inhibition became a valuable type of therapy in the clinic for the treatment of highly vascularized cancers, including glioblastoma multiforme [15].

Within the VEGF family of proteins, VEGF-A is the prototype member; the others include VEGF-B, VEGF-C, VEGF-D, VEGF-E (a virally encoded protein), svVEGF (snake venom VEGF), and placental growth factor (PlGF; also known as PGF) [16]. VEGF-A, simply known as VEGF, is the most important regulator of angiogenesis, and VEGF-A_165_ the most frequently expressed isoform (others include *VEGF*_121_, *VEGF*_189_, and *VEGF*_206_). The VEGF members exert their actions by binding to tyrosine kinase receptors called VEGFRs, which are a family of transmembrane glycoproteins that regulate processes such as gene expression, survival, growth, and proliferation. VEGFR-1 (also known as FLT-1) is a coreceptor for VEGF-A, VEGF-B, and PlGF. As a negative regulator, this receptor competitively inhibits the activation of VEGFR2 (known as KDR or Flk-1), acting as a decoy receptor. The most significant pathway triggered in physiological and pathological angiogenesis starts with the binding of VEGF-A to VEGFR2, which stimulates mitosis and chemotaxis in endothelial cells, inducing invasion and angiogenesis in solid tumors [17]. Other VEGF receptors, namely neuropilins (NRP-1 and NRP-2), may present *VEGF*_165_ to VEGFR-2, amplifying the effectiveness of VEGFR-2-mediated signal transduction [18]. These receptors, expressed in neurons, glial cells, immune cells, and endothelial cells, are transmembrane non-tyrosine kinase glycoproteins with extracellular domains that bind to semaphorins/collapsins (a family of neural axon guidance factors), which are furin-cleaved substrates located in the nerves and vessels, as well as VEGF-A [19,20]. The docking of VEGF-A to NRP-1 triggers different pathways involved in processes such as angiogenesis, nociception, and tumor development [19,20,21,22]. However, NRP-2’s physiological functions beyond chemotaxis, phagocytosis, antigen presentation in some immune cells [23], and lymphangiogenesis during development [24] are not well understood. Moreover, VEGFR3 (FLT-4) promotes lymphangiogenesis, which is essential to metastatic tumors as well, through its binding to VEGF-C and VEGF-D [25].

The VEGF axis is the basis for the dozens of anti-angiogenic drugs (as reviewed in references [3,26]) approved for therapeutic use in multiple cancer types. As an advantageous strategy, at least in earlier studies, angiogenic inhibition was used to target more genetically stable cells, such as vascular endothelial cells, rather than the more dynamic tumoral cells [9,27]. Despite the overall success of this type of therapy [28,29], its clinical application did not yield the expected outcomes, as it led to stronger hypoxic responses and poor tumoral prognoses due to the development of resistance. Currently, research is focused on the tumor microenvironment as a possible source of VEGF-A, with independent pathways mediating the resistance to VEGF-A inhibitors [30]. The angiogenesis strategy has recently been re-evaluated regarding its use for vascular tumor normalization, avoiding the over-inhibition of tumoral vessels, in order to improve drug and oxygen delivery [31]. Moreover, combinations of anti-angiogenic agents with chemotherapy or immune checkpoint inhibitors as well as multitargeted anti-angiogenic agents [32,33], are being tested as novel strategies.

This review focuses on the pathological and therapeutic aspects of the VEGF–virus interaction. We first describe the direct and indirect mechanisms by which some human pathogenic viruses perturb the VEGF levels and regulatory networks to induce respiratory diseases, as well as the mechanisms used by oncogenic viruses to induce tumor growth and metastasis. Then, we examine how viruses’ interference with VEGF signaling could be used against cancer, as exemplified by capsid immune platforms with VEGF-blocking peptides on their surfaces and by oncolytic vehicles that deliver targeted drugs including clinically validated specific anti-VEGF and VEGFR antibodies. Finally, we compile the vast literature on viral vectors expressing factors that induce or repress VEGF signaling and consider the biological tools used to effectively treat diverse vascular pathologies.

## 2. Pathogenic Viruses That Disrupt VEGF Levels

Human pathogenic viruses that perturb the vasculature commonly impact the VEGF system via mechanisms that range from the direct activation of VEGF transcription and/or post-transcriptional regulatory factors to indirect effects on mediators of the inflammatory response. The best-known examples are considered briefly here. The coronavirus disease (COVID-19) caused by SARS-CoV-2 can lead to progressive lung damage, known as severe acute respiratory syndrome (SARS), but its additional pathologies, such as hypoxemia and cytokine storms, are mainly caused by vascular dysfunction. Importantly, the VEGF levels in COVID-19 patients correlate with the severity of the disease; this is consistent with its role as a pro-angiogenic mediator that regulates endothelial changes. The disruption of the endothelial barrier occurs via several mechanisms, in which many of the proteins encoded by the SARS-CoV-2 genome have been implicated. Thus, a well-supported pathogenic role is played by the spike protein, which can bind to NRP-1, as well as by intestinal inflammation-induced VEGF in the duodenum through the activation of the Ras-Raf-MEK-ERK signaling pathway in enterocytes. Other changes in endothelial permeability have been ascribed to the nsp2, nsp5, and nsp7 proteins, which affect the expression of CD31, the von Willebrand factor, IL-6, and the tight junction proteins cadherin-5, ZO-1, and β-catenin [34,35,36,37,38,39].

Another important human pathogen, Respiratory Syncytial Virus (RSV), induces lung disease through mechanisms largely mediated by the release of active vascular cytokines. The RSV infection causes bronchial epithelial monolayer permeability via VEGF induction. Consistent with this, VEGF isoforms can be found in the nasal washings of patients with RSV infections [40,41]. Similarly, recent studies have demonstrated the relevance of the VEGF levels in the pathophysiology of the recurrent respiratory papillomatosis (RRP) of the upper aerodigestive tract caused by Human Papillomavirus (HPV) types 6 and 11 infections [42,43]. A distinct pathogenic mechanism is found in some large DNA viruses, such as the members of the *Poxviridae*, as they encode factors that are homologous to VEGF that promote vascular disruption and angiogenesis, thereby facilitating the virus’ dispersion throughout the organism [44,45,46]. Finally, other pathogenic mechanisms that are used by viruses belonging to different families, which also alter the VEGF levels, causing vascular damage or angiogenesis, are still essentially unknown (as reviewed in [47]).

## 3. VEGF Induction in Oncogenic Viruses

An extensive number of contributions over the last twenty years have revealed the important role of VEGF in the multistage development of invasive cancer [48,49]. These studies also show that, in addition to oncogenes and tumor suppressor genes, the VEGF system is involved in the mechanisms that many oncogenic viruses use to initiate the neoplastic process, promote tumor growth, or induce metastasis. Below, we briefly review several examples of oncogenic viruses that exploit the cell-signaling machinery at different stages to increase VEGF expression and angiogenesis, leading to tumor development. 

In viruses with RNA+ genomes, VEGF may also be a mediator of their oncogenic capacity. One example is the *Retroviridae*, which is one of the most extensive and well-studied virus families; it generally causes tumors through the transduction of oncogenes or activation of proto-oncogenes. Moreover, the avian leukosis virus (ALV), which causes hemangiomas and myeloid tumors in chickens, the viral gp85 envelope and p27 capsid proteins induce IL-6 gp8 via PI3K- and NF-κB-mediated signaling, which upregulates VEGF and VEGFR-2 expression in vascular endothelial cells to promote tumorigenesis [50]. The human infection caused by the RNA+ Hepatitis C virus (HCV), a member of the *Flaviviridae*, causes chronic hepatitis in a significant number of affected individuals; this gradually progresses to liver cirrhosis and subsequently to hepatocellular carcinoma. The HCV core protein upregulates VEGF and promotes angiogenesis, which is essential for tumor progression, via the kappaB/hypoxia-inducible factor-1alpha axis [51,52]. The major factor responsible for many hepatocellular carcinoma cases worldwide is the Hepatitis B virus (HBV); this is a DNA virus that also severely alters the VEGF levels via the viral protein X (HBx) that stabilizes HIF-1, enhancing hypoxia signaling during hepatocarcinogenesis [53,54,55,56]. 

Small icosahedral oncogenic viruses with double-stranded circular DNA genomes, which encode proteins that alter or degrade cell cycle regulators and tumor suppressors, also include VEGF as a target in their oncogenic mechanisms. For example, in the *Polyomaviridae* family, the multifunctional nature of the SV40 T antigen includes its ability to induce the VEGF promoter in several human malignant mesothelioma cells [57]. Viruses belonging to the *Papillomaviridae*, which represent distinct risk factors for cervical carcinoma in humans, encode early (E) proteins that are largely responsible for their oncogenic mechanisms through interactions with pRB and p53. However, through other factors or functional domains, they also dysregulate the VEGF levels in the induction and progression of carcinoma. Indeed, the E5, E6, and E7 proteins in different types of human papillomavirus (HPV) may increase the VEGF expression levels through different mechanisms to enhance angiogenesis in cervical carcinoma cells [58,59,60]. For example, the E7 protein induces VEGF through the activation of the telomerase reverse transcriptase [61], and the E6 protein activates VEGF expression through the direct stimulation of its promoter [62]. These findings on the importance of VEGF in cervical cancer are supported by the in silico design of VEGFR inhibitors as a proposed therapy via deep learning [63]. 

Large DNA viruses, such as the members of the Herpesviridae, also encode factors that perturb the VEGF levels in the associated neoplasia and in complex host interactions. In the neovascularization observed in ocular Herpes simplex-1 disease, the virus-encoded ICP4 factor increases VEGF transcription due to the similarities between its promoter and those of the virus’ early genes [64]. This is exacerbated by changes in the balance between VEGF-A and its soluble inhibitory receptor [65]. In the nasopharyngeal carcinoma caused by the Epstein–Barr virus, the latent membrane protein 1 upregulates VEGF, increasing hyperplasia and vascularization, which lead to keratoacanthoma as well as p16INK4a and matrix metalloproteinase 9 induction [66,67]. Moreover, the EBNA-1 viral antigen modulates the AP-1 transcription factor to enhance angiogenesis [68]. The Kaposi’s sarcoma-associated herpesvirus (KSHV) encodes different regulatory and invasion factors, such as ORFK13/vFLIP, K1, or vIRF3, that induce VEGF, leading to angioproliferative diseases [69,70,71,72,73]. Given the recent accumulation of reports on VEGF–virus interactions, it is expected that, in the coming years, the number of oncogenic viruses that disturb the VEGF levels through direct or indirect mechanisms will increase significantly.

## 4. Therapeutic Viral Capsids That Expose Peptides That Compete with VEGF

Viral capsids may constitute powerful therapeutic agents for pathological conditions in which the dysregulation of the VEGF system is involved. Anti-angiogenic therapies can target the VEGF system by inhibiting either VEGF itself, the VEGF–VEGFR(s) interactions, or the VEGF receptors and co-receptors, blocking their signaling. Therefore, in certain biomedical applications, virus genomes are being engineered to present VEGF-blocking peptides on their capsid surfaces, intended serve as anti-neovascularization vaccines. They are expected to induce polyclonal antibodies and provide long-term immunity against VEGF, thereby augmenting existing anti-cancer therapies. Furthermore, as discussed below, capsids engineered in precise domains may allow the development of viruses with enhanced tropism toward VEGF receptors expressing cells that mediate angiogenic processes in cancer. 

### 4.1. Peptides That Inhibit VEGF’s Functions: Binding Features and Biological Effects

**Figure 1:**
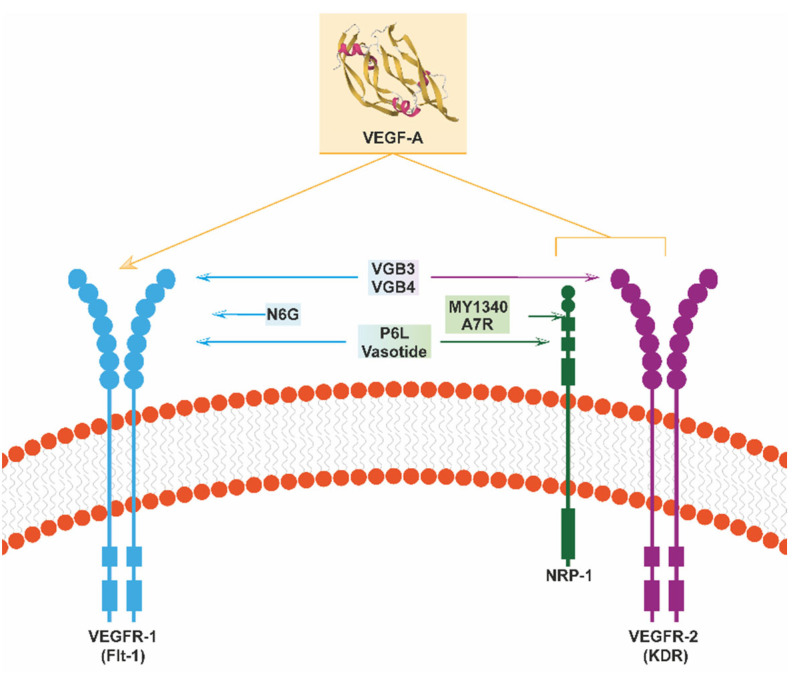
**Peptides that interfere with VEGF functions.** The figure illustrates the best-characterized peptides that bind VEGF receptors and thereby inhibit their respective signaling. See text for a detailed explanation.

As is often the case in factor–receptor interactions, the side-chain functional groups of a small set of critical residues are responsible for a substantial part of the binding affinity. VEGF mimic peptides can be designed to disrupt the interaction between VEGF and its receptors [74]. A wide range of homologous and heterologous peptides have been designed and tested for their ability to inhibit VEGF’s signaling and its capacity for neo-angiogenesis. Table 1 provides a representative list of peptides that inhibit VEGF signaling, while their interactive properties are illustrated in Figure 1, and some of their major biological properties and therapeutic effects are briefly discussed below.

In the first study using library screening for antibody binding [88], the heptapeptide A7R (V1 [ATWLPPR]) was identified [85]. A7R competes with *VEGF*165 for NRP-1 binding, potentially disrupting the VEGFR-2–NRP-1 complex and interrupting the VEGFR-2-mediated mitogenic effects and angiogenesis [86]. Molecular docking studies showed the extensive A7R-binding features. It binds two domains of VEGFR-2: one mediated through the ionic interactions of an Arg residue with Asp1054 and the other through the indole fragment of a Trp interacting with the Phe1045, Phe916, Ala864, and Leu1033 residues. With regard to NRP-1, A7R is localized at the head of the b1 domain, mimicking the C-terminal tail of *VEGF*165 and its ionic interactions with several receptor residues. In another early study that involved selection from a phage display library, the N6G (NIRRQG) peptide showed high affinity in binding to the VEGFR-1 receptor, and the P6L (PQPRPL) peptide containing the overlapping binding sites of two VEGF-B isoforms (VEGF-B167 and VEGF-B186) bound both VEGFR-1 and the neuropilin-1 receptor (NRP-1) [75].

Other interesting peptides identified in more recent studies include VGB4 (KQLVIKPHGQILMIRYPSSQLEM), a chimeric peptide overlapping VEGF-A and VEGF-B that is able to block both VEGFR1 and VEGFR2 simultaneously. This peptide exhibits anti-angiogenic properties in vitro [76] and suppressive activity regarding tumor growth and metastasis in different types of cancer [76,77,78]. VGB3 (ECRPPDDGLC) is a circular peptide that reproduces a binding region of VEGFB that can bind to both VEGFR1 [79] and VEGFR2 [80], and it shows anti-angiogenic and anti-tumor activity in vitro [79,80]. PCAIWF is an interesting peptide that is capable of selectively binding to a common pocket of the three main VEGF receptors (VEGFR-1, VEGFR-2, and VEGFR-3); it inhibited angiogenesis and retinal neovascularization in a mouse model [81]. Finally, other tested peptides that display similar binding specificity but possess particular properties are: MY1340 ([(TKPRKHG)2-K]2-K-G), which contains the C-terminal binding domain of VEGF to NRP-1 and inhibits the binding of these two proteins by interacting with NRP-1 [87]; the retro-inverted peptidomimetic compound D(LPR), which also binds to NRP-1 and VEGFR-1 and has exhibited anti-angiogenic activity in models of angiogenesis and retinopathy when administered in eyedrops [83]; and the cyclic retro-inverted peptidomimetic [D(CLPRC)] named Vasotide, which binds to VEGFR-1 and NRP-1 with higher affinity than P6L [82,83] and was effective against retinal angiogenesis in two models of retinopathy in mice and monkeys when administered in eyedrops [84]. 

### 4.2. Engineering Viral Capsids with VEGF-Blocking Peptides

**Figure 2:**
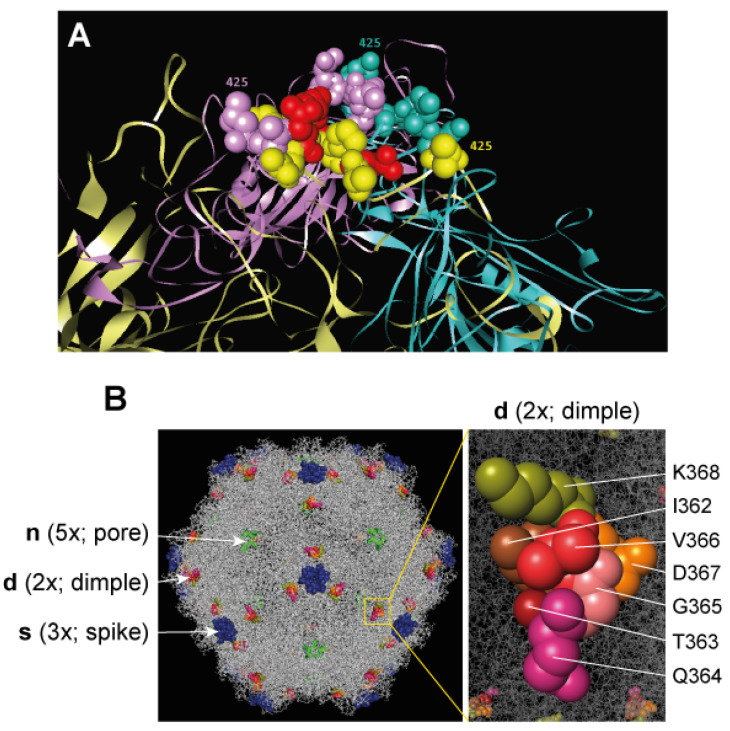
**VEGF-blocking peptides engineered via amino acid replacement at two domains of the MVM capsid.** (**A**) Replacement at loop 4 of the MVM capsid spike. The figure illustrates the amino acids residues of loop 4 replaced with VEbp in the structure of the three VP2 subunits configuring the MVM spike (reproduced from reference [99], with permission). (**B**) Domains of the MVM capsids manipulated with VEbp. The precise set of residues at the depression of the 2 × axis (named dimple) replaced with VEbp are highlighted to the right (modified from reference [100]).

Peptides with the capacity to block VEGF’s functions (VEbp) offer potential biological tools to engineer viruses with novel anti-neovascularization properties. Major attempts in this area have been performed with the Minute Virus of Mice (MVM), a member of the *Parvoviridae* [89], which is a family with 4–6 kb linear single-stranded DNA (ssDNA) with a nonenveloped icosahedral capsid of approximately 25 nm in diameter. As in many other parvoviruses, the structure of the MVM capsid, resolved to an atomic resolution [90,91], showed the VP1 and VP2 protein subunits conforming to a β-barrel of eight β-sheets, with large loops disposed between the sheets and four loops from three subunits intertwined at the three-fold symmetry axis to form the spike of the capsid (Figure 2A). By genetically engineering the parvovirus MVM to display specific peptides on the capsid surface, researchers sought to improve its tumor-targeting capabilities and oncolytic activity [92,93], as parvovirus capsids are generally effective in generating both humoral and cell-mediated immunity due to their efficacy as antigen presentation vehicles [94].

However, the compact and organized structure of the parvovirus icosahedral capsid presents significant engineering challenges, as both the functionality of the VEGF-inserted peptides and the tightly regulated nuclear assembly of infectious particles [95] need to be maintained. These restrictions have been manifested when manipulating several parvovirus capsids with heterologous peptides for immune-related retargeting applications [96,97]. The best insertion sites can be difficult to predict, as certain heterologous peptides inserted at the highly exposed loops of the capsid may disrupt the assembly [92], while others may be tolerated [93], and even disordered domains may not be conducive to insertion as they play complex mechanistic roles in virus infectivity. For example, inserting the anti-angiogenic A7R peptide into the flexible N-terminal domains of capsid proteins resulted in assembled but noninfectious DNA-filled MVM particles due to the failure of the crucial post-entry VP2-Nt cleavage necessary to initiate infection [98].

Consequently, other MVM capsid domains with different functional requirements were explored, and the substitution of viral residues with VEbp, rather than their mere insertion, was considered. Two domains of the capsid were targeted using this amino acid replacement approach. In the first study of peptide replacement, the loop 4 contributing to the spike at the three-fold axis of the capsid (Figure 2A), a major immunodominant domain [101], was targeted. The infectious chimeric MVM viruses exposing the A7R and P6L peptides on loop 4 of the spike were viable; they were able to induce anti-VEGF antibodies without an adjuvant as well as to evade neutralization by MVM-specific antibodies [95]. However, the manipulation at the spike imposed structural restraints that disturbed both the VEbp configuration and capsid assembly, which the virus could overcome via evolution; this restored the assembly functions but modified the VEbp sequence [95]. In the second study, the amino acid residues at three MVM capsid functional domains were replaced with the A7R, P6L, and N6G peptides, attempting to retarget the infectious virus to the tumor vasculature [102]. Although most substitutions impaired virus maturation, the replacement of the residues with VEbp in a dimple at the two-fold axis that drove MVM’s tropism through sialic acid (sia) binding altered the viral tropism (Figure 2B). Meanwhile, retargeting to the VEGF receptors failed due to the structural constraints imposed by the narrow dimple on the VEbp configurations. The chimeric viruses showed increased tropism to tumor cells expressing different types of α2-3,-6,-8-linked sia receptors. Nonetheless, this study shows the usefulness of VEbp insertions to retarget oncolytic viruses to sia types, triggering viral entry into tumor cells. 

## 5. Anti-VEGF Antibodies: From Development to Clinical Application

**Figure 3:**
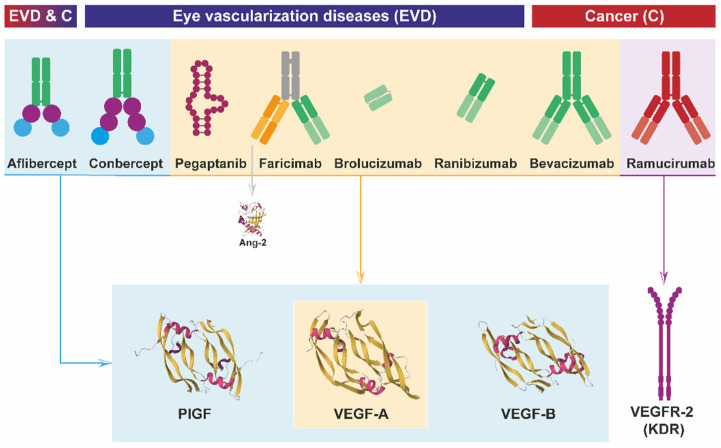
**Macromolecules that interfere with VEGF’s functions.** Approved biomolecules that compete with the VEGF system are illustrated. Upper: Antibodies, fusion proteins, and aptamers with therapeutic applications in eye vascularization diseases (EVD), cancer (C), or both (EVD and C). The respective bound VEGF factor or receptor is outlined. Lower: The structures of three targeted VEGF family members and VEGFR-2.

As anti-angiogenic agents that are of great interest at present, many virus vectors are being developed; in some cases, they are being applied in clinical trials, expressing the gene of an antibody interfering with the VEGF system. The following is a brief description of the major antibodies sharing this remarkable anti-VEGF property, and their expression from virus vectors to application in diverse vascular therapies is described in the sections below. Indeed, VEGF antagonists were quickly proposed as potential therapies for the treatment of cancer and other angiogenic disorders [103]. It was demonstrated that specific monoclonal antibodies could block VEGF-induced angiogenesis both in vivo and in vitro, providing the first evidence that the targeting of a paracrine mediator affecting the vasculature could significantly inhibit tumor growth. VEGF inhibition could thus be therapeutically valuable for the treatment of highly vascularized and aggressive cancers [13]. Several other monoclonal antibodies were soon described and tested for anti-VEGF purposes [104,105], such as inhibiting VEGF-induced endothelial cell proliferation in vitro, as well as tumor growth [106], and they were tested for their safety in clinical trials [107]. Anti-angiogenic therapy regarding the VEGF system can target VEGF itself or inhibit the VEGF receptors and co-receptors mediating the intracellular signaling pathways. Currently, seven VEGF/VEGFR interaction inhibitors have been approved by the FDA and EMA, with one additional approval by the National Medical Products Administration (NMPA) in China; all are mentioned hereafter. As illustrated in Figure 3, three of these inhibitors were approved for cancer treatment as anti-angiogenic drugs, whereas six were approved to treat ocular hypervascularization diseases [100].

**Figure 4:**
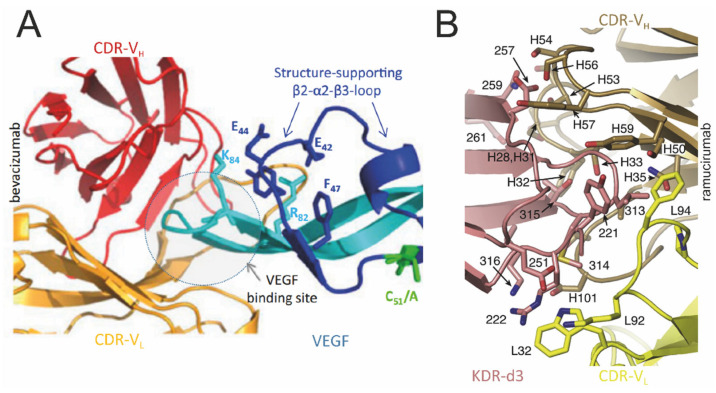
**Structures of VEGF–antibody complexes.** (**A**) The structure of the binding domain of bevacizumab to VEGF. The figure illustrates the antibody bevacizumab as a light chain (orange) and heavy chain (red), binding to the VEGF β5–turn–β6 loop (light blue), in a ball-and-stick representation (reproduced from reference [110], with permission). (**B**) The KDR d3–ramucirumab interface in detail. The backbones of KDR and ramucirumab are shown in a colored cartoon representation as follows: KDR d3 (pink), the ramucirumab light chain (yellow), and the ramucirumab heavy chain (brown). The side chains involved in the KDR–ramucirumab interactions are shown in stick form. Numbers only identify KDR residues, ramucirumab heavy-chain residues are denoted by the prefix “H”, and light-chain residues are denoted by the prefix “L” (reproduced from reference [119], with permission).

The humanized anti-VEGF compound bevacizumab (Bvz; Avastin) was the first monoclonal antibody designed for anti-angiogenic therapy; it was approved by the FDA in 2004 [108]. Bvz targets VEGF-A, preventing its interaction with VEGFR and therefore inhibiting this signaling pathway that triggers angiogenesis. Treatment with Bvz leads to a reduction in the microvasculature in tumor blood vessels [109], and in combination with chemotherapy, it brings clinical benefits in the treatment of different tumors while exhibiting low toxicity [28]. The resolution of the crystal structure (Figure 4A) provided insights into the key residues that affect the VEGF-Fab region of the interaction at the outer strands β-5 and β-6 of VEGF. The interface between VEGF and the Fab fragment involves 25 residues from the Fab fragment and 19 residues of VEGF, with the segment from Met81 to Gly92 being essential for recognition and binding to the antibody [110]. A therapeutic vaccine using hVEGF26-104, the minimal peptide that binds with high-affinity bevacizumab [111], was considered safe and well tolerated among cancer patients. However, no clinical benefit was observed as the treatment failed to induce seroconversion against the native hVEGF_165_ [112], despite inducing VEGF_165_-neutralizing antibodies in a non-human primate model [113]. 

The first monoclonal antibody drug targeting the VEGFR-2 receptor was ramucirumab (Cyramza; [114]), which was approved by the FDA in 2014. Unlike other agents targeting the VEGFR-2/VEGF pathway, ramucirumab attaches to a specific site in the extracellular domain of VEGFR-2, effectively preventing all identified VEGF ligands from interacting with this target [115,116], thereby preventing VEGFR-2’s phosphorylation and inhibiting the downstream signaling pathways that lead to angiogenesis [117]. Whether used alone or alongside chemotherapy, ramucirumab has shown considerable benefits in pretreated cancer patients [118]. In the crystal structure analysis (Figure 4B), ramucirumab’s binding epitope was found to be localized at the short β-hairpin β6-β7, an adjacent section of the β-strand and the N-terminus of domain 3. The functional domain was found to be the region between the Gly312 and Lys316 residues [119].

Other antibodies that are worth mentioning are ranibizumab, an affinity-matured bevacizumab-derived Fab antibody with increased binding affinity to VEGF-A [120]; brolucizumab, a humanized monoclonal single-chain FV (scFv) antibody fragment that targets VEGF-A with the same affinity as ranibizumab [121,122]; and Faricimab, a bispecific antibody composed of one Fab as ranibizumab and an anti-Ang-2 and modified Fc region [123].

Finally, besides antibodies, some molecules have shown effective anti-angiogenic activity, such as Pegaptanib, which is a 28-base ribonucleic acid aptamer that binds specifically to the 165-amino-acid form of VEGF-A [124,125]; aflibercept, a homodimeric fusion protein consisting of the Fc region of human IgG1 combined with the second domain of VEGFR1 and the third domain of VEGFR2 [126], with high binding affinity to VEGF-A, VEGF-B, and PIGF [127]; and the protein Conbercept, containing the Fc part of human IgG1 bound to the extracellular domain 2 of VEGFR1 and the extracellular domains 3 and 4 of VEGFR2 [128], as well as binding VEGF-A, VEGF-B, and PIGF [129].

It should be mentioned that although passive immunotherapy treatments with antibodies that bind VEGF or its receptors are widely applied in modern cancer therapy, their long-term safety remains under debate due to the adverse effects experienced by cancer patients in their healthy vasculature across various tissue types and organs [130]. Therefore, novel effective vaccines are being developed based on peptides that mimic the binding sites and signaling mechanisms of VEGF and its receptors; these minimize the off-target effects and associated toxicities, leading to better clinical outcomes for cancer patients [111,131,132]. In this context, such anti-angiogenic specific peptides, particularly those recognized by approved antibodies, may in turn allow therapeutic vaccines to be delivered on chimeric viral capsids, as described above. 

## 6. Oncolytic Viruses as Anti-Angiogenic Agents

The use of oncolytic viruses (OV) that preferentially replicate in and kill tumor cells without significant effects on healthy cells has been extensively explored as therapeutic agents in the last few decades, considering multiple cancer types. However, in many cases, OV clinical trials have revealed limited virus spread with insufficient therapeutic outcomes, resulting in tumor resistance or relapse, which underlines the need for the reinforcement of OV-based anti-tumor therapies, as outlined below. 

### 6.1. OV with Natural Anti-Angiogenic Properties

Some OV present anti-angiogenic properties that contribute to their pathogenicity and anti-cancer capacity. For example, a vaccinia virus (VV) therapy showed a critical delay in re-vascularization, which was re-established only after viral clearance, suggesting a direct anti-angiogenic effect of VV [133]. Likewise, oncolytic adenoviruses decreased the expression of HIF-1alpha and VEGF, resulting in combined anti-angiogenic and immune-stimulatory effects against cancer [134]. Another example was the intravascular administration in a syngeneic mouse model of ovarian carcinoma of a replication-restricted oncolytic herpes simplex virus type 1 (HSV), showing tumor cytotoxicity with potent anti-angiogenic effects in vivo, derived from a selective infection of the tumor endothelium [135]. Similarly, the intravenous administration of vesicular stomatitis virus (VSV) in a CT-26 syngeneic murine model of colon cancer reduced the proliferation of tumor cells via the specific infection and destruction of the tumor vasculature, without affecting the normal vasculature [136]. These reports illustrate that some OV provide dual anti-cancer effects based on the direct infection of tumor cells and also on the modification of the pro-angiogenic environment necessary for tumor growth and dissemination.

### 6.2. OV in Combined Anti-Angiogenic Therapies

Therapies combining OV with anti-angiogenic factors, with or without immunotherapy, have been considered in the search for definitive treatments for cancer, with promising outcomes (as reviewed in [137,138,139]). Among RNA viruses, an oncolytic reovirus combined with VEGF165 inhibitors promoted lysis in mouse melanoma cells and triggered an innate immune-mediated attack on the tumor vasculature, resulting in long-term anti-tumor effects [140]. Likewise, the combined administration of the negative RNA vesicular stomatitis virus (VSV) with the receptor tyrosine kinase inhibitor (RTKI) sunitinib resulted in an improved, sustained anti-tumor effect in diverse prostate, breast, and kidney tumor mouse models as compared to monotherapies [141].

Among DNA viruses, replication-selective adenovirus E1ADeltaCR2 (dl922-947) combined with bevacizumab improved the viral distribution and effect in xenograft mouse models of human thyroid anaplastic carcinoma [142] and glioblastoma [143]. Among the large DNA viruses, significant therapeutic effects have been obtained with natural and genetically engineered herpesviruses. For instance, anti-VEGF antibodies may enhance the anti-tumor effects of HSV virotherapy via their anti-angiogenic effects and the modulation of the host inflammatory reaction against the virus [144], or they may enhance viral distribution as well as tumor hypoxia, achieving synergistic anti-breast cancer effects [145], although their administration prior to that of the virus may inhibit the tumor infection [138]. Similarly, an HSV-1 herpesvirus overexpressing vasculotatin (an extracellular fragment of a brain-specific angiogenesis inhibitor, BAI-1), administered in combination with bevacizumab, increased the survival rate in glioma xenograft murine models (as reviewed in [146]). Moreover, propranolol, a nonselective beta-blocker with anti-angiogenic effects, also showed synergistic effects when combined with HSV in a colorectal tumor murine model [147]. Other large oncolytic DNA viruses, such as the JX-594 poxvirus, expressing the granulocyte–macrophage colony-stimulating factor (GM-CSF) and designed to selectively replicate in cancer cells, yielded a superior effect when administered sequentially with sorafenib (a VEGFR inhibitor) in patients with hepatocellular carcinoma [148]. These reports suggest that an enhanced anti-tumoral effect can be achieved in combined treatments, although the timing of the administration of the OV and anti-angiogenic factors may be critical to obtain the optimal results, as anti-angiogenic factors can negatively affect the efficiency of viral infection.

### 6.3. OV Expressing Anti-Angiogenic Factors

The development of OV-expressing anti-angiogenic factors is an active area of research in the pursuit of more efficient anti-tumoral treatments. Table 2 provides a summary of several reports based on different OV, which are discussed briefly here.

In the field of adenoviruses, the inoculation of an E1B-55-kDa-deleted oncolytic adenovirus (ZD55) armed with a soluble form of VEGFR1 led to a marked reduction in colon tumor growth with a decreased microvessel density and increased tumor apoptosis [149], whereas similar adenovirus vectors provoked enhanced anti-angiogenic and anti-tumor effects in colon and prostate xenografts murine models [150], with reduced vessel densities and increased apoptosis in both endothelial and tumor cells [152]. Other adenoviruses expressing a soluble form of VEGFR1 reduced the growth rate of human breast cancer [151], as did an adenovirus expressing a humanized anti-VEGF monoclonal antibody [153]. A non-replicative adenovirus overexpressing a soluble form of VEGFR2 caused tumor remission in a breast cancer murine model [133]. Adenoviruses expressing other anti-VEGF elements, such as short hairpin RNA (shRNA), an artificial transcription factor based on zinc-finger proteins (ZFPs) to target VEGF promoters, or a PD-L1 shRNA, have also shown enhanced anti-tumor effects through diverse mechanisms [154,155,156].

Some large OV armed with anti-VEGF elements that are worth mentioning include the vaccinia virus (VV) armed with GLAF-1 (a single-chain antibody that is directed against human and mouse VEGF), which showed high activity in diverse subcutaneous human tumor xenograft murine models [157]. Moreover, a double-deleted VV that replicated exclusively in cancer tumor cells was armed with a soluble form of VEGFR1 and triggered the activation of the innate immune response and the high expression of cytokines, mediating a strong anti-tumor effect [158]. A VV encoding a single-chain antibody against VEGF showed tumor anti-angiogenic effects in a murine orthotopic model of triple-negative breast cancer [159]. Additionally, in a combined treatment with radiotherapy against a subcutaneous glioma, it provoked the radio-sensitization of the tumor-associated vasculature and caused tumor shrinkage [160]. The simultaneous expression of three antibodies in a single VV led to enhanced tumor regression, suggesting that the interference of more than one tumor growth-stimulating mechanism results in enhanced therapeutic efficacy against cancer [161]. Another large OV, the HSV, was genetically engineered to express angiostatin (an endogenous inhibitor of angiogenesis) and bevacizumab, and it was applied in a human glioblastoma mouse model and showed tumor lysis and angiostatin-mediated VEGF inhibition, suppressing invasive phenotypes with higher survival time values [162]. Finally, the small rodent parvovirus H-1PV, which presents intrinsic oncotropism and a good safety profile for use in cancer virotherapy, caused the inhibition of VEGF expression inhibition in Kaposi’s sarcoma cells when chemokine-armed [163]. In summary, the results obtained in preclinical assays with diverse virotherapies and combined anti-angiogenic treatments suggest that strong synergistic anti-tumor effects can be achieved, which deserve exploration in clinical assays, ideally supported by immunotherapy to prevent tumor relapse and resistance. 

## 7. Anti-Angiogenic AAV Vectors in Cancer Therapy

A group of viruses that is worth mentioning because of their extensive use as anti-cancer vectors that interfere with VEGF’s functions are the various serotypes of the human adeno-associated parvovirus (AAV). Although the natural oncolytic capacity of these ssDNA viruses is low because they are not self-replicating infectious viruses, they have unique properties, such as low pathogenicity in humans, and extensive knowledge has been accumulated in basic research on their mechanisms of genome regulation, which allows their efficacy and biosafety to be optimized. Thus, multiple anti-tumor factors have been delivered in cancer therapies using AAV vectors, including cytotoxic and anti-angiogenic molecules (as reviewed in [164,165]). See Table 3 for a summarized description of the most widely studied anti-angiogenic AAV vectors with applications in cancer therapy. 

The first assays used VEGF modulators expressed in AAV viral vectors. For example, rAAV serotype 2 encoded a soluble form of VEGFR2 in murine models of pediatric kidney tumors and metastatic neuroblastoma, yielding the significant inhibition of tumor growth and metastasis [167,168]. Other similar rAAV vectors expressing soluble forms of VEGFR1 and a combination of VEGFR1 plus VEGFR2 were also assayed in ovarian tumors and glioblastoma, with beneficial effects [166,169,173]. Notably, the overexpression of a soluble form of the VEGFR3 receptor involved in the stimulation of VEGF-C-mediated tumor-associated lymphangiogenesis via a rAAV serotype 8 vector resulted in the inhibition of lymph node metastasis in murine models of melanoma, kidney, and prostate tumors [174]. 

In parallel strategies to combat cancer, several rAAV vectors expressing antibodies against VEGF have been developed. For instance, bevacizumab delivered to the lung or peritoneum or intracranially via an rAAV serotype rh.10 reduced lung metastasis, tumor growth, and vascularity in metastatic prostate carcinoma, as well as tumor growth and angiogenesis in ovarian cancer and glioblastoma [176,177,178]. The VEGF-Trap soluble decoy receptor (containing the second Ig-like domain of VEGFR1 and third Ig-like domain of VEGFR2 fused to a human IgG Fc) delivered via an rAAV serotype 2 through intravenous administration reduced not only the concentration of VEGF in the serum but also primary tumor growth, preventing pulmonary metastasis in breast cancer [170]. 

In more recent trials, rAAV vectors expressing anti-angiogenic factors have been administered in combined therapies, attempting to reinforce their anti-tumoral effects in preventing tumor resistance or relapse. Hence, the AAV2-VEGF-Trap vector was used in a chemotherapy combination for the treatment of triple-negative breast cancer (TNBC) in a xenograft mouse model. The apparent diffusion coefficient (ADC) of the vector was analyzed via diffusion-weighted magnetic resonance imaging (DW-MRI). A single injection of this vector, combined with paclitaxel, resulted in synergistic effects against TNBC growth and neovascularization [171]. The same AAV2-VEGF-Trap vector was assayed in combination with temozolomide (TMZ) or bevacizumab in a murine glioma model and synergistically decreased the microvascular density and enhanced tumor apoptosis [172]. Finally, AAV serotype 8 vectors overexpressing both soluble VEGFR2 and VEGFR3 receptors combined with paclitaxel and carboplatin resulted in strong anti-angiogenic and anti-tumor effects in the treatment of ovarian cancer [175].

## 8. Therapeutic AAV Vectors Targeting the VEGF System

Vascular disorders are commonly treated using pharmacological pro-/anti-angiogenesis therapies, seeking to recover normal vascular function. However, in the last decade, these treatments have been substituted with gene therapy strategies, attempting to achieve more sustained effects. These new therapies require efficient and safe therapeutic vectors, with those derived from the parvovirus adeno-associated serotypes (rAAV) being the most widely used for in vivo therapies. Several characteristics make rAAV an ideal vector for these applications, such as their stable long-term transgene expression, capacity for the transduction of dividing and non-dividing cells, low insertional capacity that minimizes possible insertional mutagenesis, and low immunogenicity. Hence, rAAV have become the preferred gene transfer vectors in pro-angiogenic or anti-angiogenic clinical therapies (as reviewed in [165,179,180,181]). We review a number of diseases related to VEGF functions in which therapies using rAAV vectors were successfully applied.

### 8.1. Pro-Angiogenic rAAV Vectors

Viral vectors expressing pro-angiogenic agents were initially constructed in an attempt to generate animal models of angiogenesis processes. Following this approach, the intravitreal injection of VEGF-overexpressing rAAV vectors allowed the analysis of the VEGF-A-mediated effect on the transcriptome in the retina and the posterior layer of the eye. The transduction yielded similar clinical effects to those obtained in oxygen-induced retinopathy murine models, confirming the main role of VEGF in these pathologies (as reviewed in [182]). While adenovirus vectors provided animal models for short-term choroidal neovascularization (CNV) studies [183], VEGF-expressing AAV vectors permitted long-term murine models of age-related macular degeneration (AMD) [184]. Similarly, an AAV ShH10 variant vector overexpressing VEGF in the form of Müller glia-specific expression was used to generate murine models of retinopathy of prematurity (ROP) and oxygen-induced retinopathy (OIR) [185]. 

In therapies for diverse pathologies, VEGF-expressing rAAV vectors are being extensively assayed (see Table 4). For example, in ischemic stroke rodent models, a methacryloyl gelatin microneedle (GelMa) was used for the sustained and controlled release of VEGF expressed in an rAAV vector; upon homogeneous and strong transduction, this resulted in augmented angiogenesis and neurogenesis [186]. In another study, the overexpression of VEGF-C from an rAAV serotype 8 vector was used for the treatment of hepatic encephalopathy [187], and a serotype 9 vector was used for the treatment of ischemic stroke in a murine model, in which the vector promoted the drainage of the cerebrospinal fluid, neurogenesis, and protection [188]. Recently, rAAV serotype 9 vectors overexpressing VEGF-A have been used for the treatment of spinal cord injuries, showing locomotor function recovery and tissue damage protection, but only when the vector was administered prior to the injury [189]. In a recently assayed combined treatment, an rAAV serotype 9 vector expressing a combination of two vasculogenic factors (VEGF and PDGF) showed promising therapeutic effects on bone pathologies in Yucatan mini-pigs with tibial diaphyseal defects, although vascular lesions with excessive bleeding were developed [190].

### 8.2. Anti-Angiogenic rAAV Vectors

The intravitreal injection of the anti-VEGF antibodies and molecules mentioned above (e.g., bevacizumab, ranibizumab, aflibercept, conbercept, and brolucizumab) is being recurrently applied for the prevention of severe vision loss in patients with ophthalmologic pathologies. However, repetitive injections may cause intraocular inflammation, retinal detachment, or ocular hemorrhage, with poor results. Accordingly, viral vectors overexpressing anti-angiogenic factors are emerging as alternative therapies to avoid these repetitive, harmful administrations. Indeed, almost thirty percent of the current rAAV gene therapy clinical trials are focused on eye diseases, as the administration of rAAV via subretinal and intravitreal injection results in a safe and effective treatment. Some major contributions are listed below on the basis of the nature of the transduced inhibitor (see Table 5).

In AMD murine models, multiple rAAV overexpressing diverse anti-angiogenic therapeutic factors (anti-VEGF antibodies, anti-VEGF small hairpin RNA (shRNA), and others) are being used (as reviewed in [33,181] (https://clinicaltrials.gov/, accessed 23 July 2024). For example, the subretinal administration of AAV serotype 8 overexpressing a single-chain fragment variable (ScF) antibody against VEGF prevented angiogenic effects in a laser-induced CNV murine model with non-significant toxicity [180]. 

When using rAAV-expressing protein decoys, the results were particularly impressive with the aflibercept fusion protein (see above) in the AAV 2.7m8 vector (ADVM-022), modified for retina transduction, which maintained robust expression even after a year of post-intravitreal administration in laser-induced CNV primate models. Indeed, ADVM-022 is currently being applied in phase 2 clinical trials evaluating its safety and efficacy for the treatment of diabetic macular edema (DME) and wet and neovascular AMD as well as in a long-term follow-up study on DME (as reviewed in [191]; https://clinicaltrials.gov/, accessed 23 July 2024; NCT04418427, NCT03748784, NCT05536973, NCT05607810, respectively). Similarly, conbercept (see above), when overexpressed in diverse rAAV serotypes and given via intravitreal administration, reduced the number of retinal aneurysms in OIR and prevented choroidal neovascular lesions in a laser-damaged mouse model, although the transduction efficiency depended on the serotype (as reviewed in [192]).

**Table 5:** AAV vectors expressing anti-VEGF factors.

Vector	Transgen	Assay	Results	References
AAV2 ^a^	shVEGF + IGF	Rat model of lumbar disc degeneration	Reduction of disc cell death in the vertebral pulp and annulus fibrosus	[193]
AAV2 ^b^	VEGF inhibitor (VID), complement inhibitor (CID) and dual inhibitor (ACVP1)	Mice models of endotoxin-induced uveitis, autoimmune uveoretinitis, and CNV	Improvement of laser induced injuries and CNV ^c^ and ACVP1 protection against ocular inflammation and neovascularization	[194]
AAV 2.7m8 (ADVM-022) ^b^	Aflibercept (fusion protein made of the VEGFR1 and VEGFR2 extracellular domains, and the Fc of the human IgG1)	Single IVT ^d^ administration in non-human primates.	Long term efficacy against grade IV lesions in CNV ^c^ models. Safety and efficacy for wet and neovascular-AMD (phase 1), and for DME and diabetic retinopathy (phase 2).	[191,195,196,197]
AAV2, ADVM-022, AAV3b, and AAV8 ^b^	Conbercept (KH902), recombinant protein with multiple Ig domains of VEGFR1 and VEGFR2	IVT ^d^ administration in mice models of oxygen-induced retinopathy and laser induced CNV	Long-term efficacy. Reduction in retinal aneurysms	[192]
AAV5 ^b^	Dual anti-VEGF-A miRNAs + PEDF	SR ^e^ administration in mouse model of CNV	Reduction of CNV^c^ area and in VEGF expression.	[198]
AAV8 ^b^	Anti-VEGF single-chain variable fragment	Mouse model of CNV	Long-term safe and effective effects	[180]
AAV8 ^b^	VEGF Trap (nVEGFi)	SR ^e^ administration in mouse model of CNV	Increased reduction in the CNV ^c^ area, and reduction of toxicity comparing with AAV8-aflibercept	[199]
AAV8 ^b^	miR-aghsRNA against VEGF	SR ^e^ administration in mouse model of CNV	Reduction of CNV ^c^ area, and no clinical signs of intra-ocular inflammation	[200]
AAV8 ^b^	CRISPR-based VEGF-A suppression	SR ^e^ administration in mouse model of CNV	Partial gene disruption and partial reduction of CNV ^c^	[201]

^a^ Vector used for therapy of lumbar disc degeneration; ^b^ Vectors used for therapy of age-related macular degeneration (AMD) and diabetic macular edema (DME); ^c^ CNV, choroidal neovascularization; ^d^ IVT, Intraviteal; ^e^ SR, Subretinal.

Concerning VEGFR expression, the intravitreal administration of an rAAV serotype 2 vector overexpressing a soluble form of VEGFR2 (1-3 Ig-like domains of VEGFR2 fused to human IgG Fc) caused the downregulation of VEGF in laser-induced CNV, although it generated mild to moderate inflammation [202]. The VEGF-Trap nVEGFi (a soluble decoy VEGFR containing the first and second Ig-like domains of VEGFR1 and the third Ig-like domain of VEGFR2 linked to human IgG Fc), expressed in an rAAV serotype 8, showed powerful transduction and therapeutic effects in retinal pigment epithelium (RPE) cells and photoreceptors [199]. 

In nucleic acid-based technologies, the RNA interference against VEGF was assayed in a porcine model of CNV, demonstrating safety and robust anti-angiogenic effects [200]. The Clustered Regularly Interspaced Short Palindromic Repeats (CRISPR) technology, applied through rAAV vectors, has been used to reduce the VEGF expression in diverse eye diseases. This technology has also been assayed in other rAAV vectors with a nuclease of *Lachnospiracea bacterium* (LBCpf1) to reduce the expression of VEGFR2 and HIF1-alpha (as reviewed in [201]). 

Combined therapies are being considered to prevent anti-VEGF resistance to monotherapy treatments. An rAAV serotype 2 engineered to secrete the ACVP1 dual inhibitor (containing a chimeric VEGF inhibitor domain VID and a complement inhibitor domain CID) attenuated inflammation in endotoxin-induced uveitis and experimental autoimmune uveoretinitis, and it showed a reduction in retinal structure damage. Dual ACVP1 vector provided significantly better protection than monotherapy vectors in laser-induced retinal and choroid/RPE injuries in CNV murine models [194]. Likewise, rAAV serotype 5 has been assayed in AMD with the combined expression of anti-VEGF-A microRNAs (miRNAs) and the pigment epithelium-derived factor under the control of a retinal pigment epithelium-specific promoter in vitelliform macular dystrophy 2. The reduction in CNV and VEGF expression in laser-induced CNV models was larger in the dual therapies than in monotherapies [198]. Finally, dual rAAV vectors are also being used in the treatment of other pathologies related to angiogenic disorders, such as lumbar disc degeneration (LDD). IGF1 deficiency and VEGF overexpression were corrected in LDD patients through the administration of rAAV vectors co-expressing IGF1 and a shRNA against VEGF, reducing the rate of disc cell death in the vertebral pulp and annulus fibrosus and increasing the levels of proteoglycans and type II collagen [193].

## 9. Concluding Remarks

We have reviewed the fascinating field that combines the complex biology of viruses with the multiple roles of the VEGF system in homeostasis and various pathologies. The main conclusion of this work concerns the multiple pathogenic mechanisms that viruses use to perturb VEGF expression and signaling in cancer and other vascular pathologies. Based on this mechanistic connection between angiogenesis control pathways and viral pathogenesis, which operates in both vascular damage and oncogenesis, future therapies may be conceivable in which anti- or pro-angiogenic drugs with the ability to normalize the physiological VEGF levels behave accordingly as antiviral agents that inhibit the progression of pathogenic viruses.

This review highlights the enormous development and therapeutic potential of multiple infectious viruses and viral vectors that, using very different strategies, can regulate the altered VEGF levels in multiple pathologies, with particular relevance in cancer and those associated with ocular vascularization. For example, some vectors aimed at VEGF inhibition exploit the well-known and extensive clinical use of specific anti-VEGF antibodies. These anti-neovascularization vectors express anti-VEGF antibodies, although sustained and transient expression may require personalized adjustments to satisfy patients’ therapeutic needs. Other types of vectors that modulate the VEGF responses employ icosahedral viruses as platforms for VEGF-blocking peptides, whose immunogenicity and tropism determinants can be exposed on the capsid surface, although the preservation of their immunogenic configuration and their genetic stability are major concerns that need to be thoroughly evaluated before their clinical application.

Other natural viruses and engineered vectors are being extensively explored as oncolytic agents against the VEGF-mediated neovascularization processes needed for cancer progression. Likewise, human AAV serotypes are being extensively developed and applied in some cases as pro- or anti-angiogenic vectors to treat vascular ocular diseases. These viruses and vectors, used alone or in combination, are endowed with factors designed to directly or indirectly target VEGF’s functions and signaling at many levels. Their specificity, efficacy, and biosafety, together with other aspects such as stability and large-scale production, will determine their eventual therapeutic capacity in the oncologic and ophthalmologic setting. Ongoing and future studies on VEGF–virus interactions will yield key insights into the complexity of both the virus biology and the VEGF system. These are expected to provide sophisticated therapeutic tools to treat angiogenesis in cancer, eye diseases, and many other VEGF-mediated diseases.

## Figures and Tables

**Table 1:** Peptides inhibiting VEGF functions.

Name	Sequence	Binding	Features	Results	Refs
N6G	NIRRQG	VEGFR1	Selected from a random phage display library	Less efficient binding than P6L	[75]
VGB4	KQLVIKPHGQILMIRYPSSQLEM	VEGFR1 and VEGFR2	Chimeric and overlapping peptide of VEGF-A and VEGF-B	Inhibition of tumor growth, metastasis and signaling patways	[76,77,78]
VGB3	ECRPPDDGLC	VEGFR1 and VEGFR2	Circular peptide reproducing VEGF-B	Anti-angiogenic and anti-tumor activity	[79,80]
PCAIWF	PCAIWF	VEGFR-1, VEGFR-2, and VEGFR-3	Selected from a random phage display library	Inhibition of tube formation and retinal neovascularization	[81]
P6L	PQPRPL	VEGFR1 and NRP-1	Overlapping peptide of VEGF-B_167_ and VEGF-B_186_	The RPL peptide is essential for the binding	[75,82]
Vasotide	D(CLPRC)	VEGFR1 and NRP-1	Cyclic peptide of retro-inverted peptidomimetic RPL from P6L	Anti-angiogenic activity in retinopathy models and inhibition of tumor growth	[83,84]
A7R	ATWLPPR	NRP-1	Mimicks VEGF-A_165_ structure	Decreased VEGF-mediated epithelial cell proliferation and tubular formation in vivo	[85,86]
MY1340	[(TKPRKHG)2-K]2-K-G	NRP-1	VEGF c-terminal polypeptide	Anti-tumor activity in vivo	[87]

**Table 2:** Oncolytic viruses expressing anti-VEGF factors.

Oncolytic Virus	Transgen	Experimental Assay	Results	References
Adenovirus (ZD55)	Soluble VEGFR1	IT * administration in xenograft subcutaneous tumor mouse model of SW620 colon cancer	Reduction in tumor growth and angiogenesis. Synergetic effect with 5-FU chemotherapy	[149]
Replication competent and deficient Adenovirus	Soluble VEGFR2	IV ** injection in subcutaneous xenograft tumor mouse model of HCT 116 colon and PC-3 prostate cancer	Enhanced anti-tumor effect in coinfection treatments	[150]
Adenovirus (AdEHE2F)	Soluble VEGFR1	IT * and subcutaneous administrations in murine models of ER-positive/negative breast cancer tumors.	Enhanced anti-tumor effect in ER-negative tumor	[151]
Adenovirus (RdB)	Soluble VEGFR3	IT * administration in subcutaneous xenograft mouse model of lung carcinoma tumor (H460)	Improved antiangiogenic and antitumor effects	[152]
Adenovirus (enadenotucirev)	Anti-VEGF antibodies (NG-135)	IV ** injection in subcutaneous xenograft mouse model of A549 lung cancer tumor	Tumor burden reduction	[153]
Incompetent Adenovirus (Ad B7and Ad-E1)	VEGF shRNA	IT * administration in xenograft mouse model of U343 glioblastoma	Reduction in tumor growth and angiogenesis	[154]
Adenovirus Ad-B7-KOX	Zinc finger protein (ZFP) against VEGF promoter	IT * administration in subcutaneous xenograft mouse model of U87 human glioblastoma	Reduction in angiogenesis increasing tumor apoptosis and survival	[155]
Recombinant Adenovirus RCAd derived from Ad5	Anti-VEGF antibody and PD-L1 shRNA	IT * administration in mouse models of subcutaneous xenograft and humanized immune system of U87 human glioblastoma.	Reduction of VEGF-A level and immune activation	[156]
Adenovirus + Vaccinia virus	Soluble VEGFR2	IV ** injection in subcutaneous xenograft mouse model of breast cancer tumor (MDA)	Tumor remission upon AdV-VEGFR2 administration after VV	[133]
Replication-competent Vaccinia virus (GLV-1h68)	Single-chain (sc)anti-VEGF antibody (GLAF-1)	Single IV ** injection in subcutaneous xenograft mouse models of human tumors (DU-145 and A549)	Higher oncolytic activity than antibody monotherapy	[157]
Double deleted Vaccinia viruses	VEGFR-1-Ig	IT * administration in subcutaneous xenograft immunocompetent mouse model of kidney cancer (786-O).	Higher antitumor and antiangiogenic effects with reduced cytokine response	[158]
Vaccinia virus (GLV-1h164)	scAnti-VEGF (GLAF-2)	IT * administration in orthotopic xenograft mouse model of TNBC tumor (MDA-MB-468)	Tumor regression and anti-angiogenic effect	[159]
Replication-competent Vaccinia virus (GLV-1h68)	(sc)Anti-VEGF antibody (GLAF-2) and radiation	Retro-orbital injection in subcutaneous xenograft mouse model of human U87 glioblastoma	Increased viral replication and oncolysis. Anti-angiogenic effects and high radio-sensitivity	[160]
Replication-competent Vaccinia virus (GLV-1h68)	Anti-VEGF, anti-EGFR, and anti-FAP antibodies	Retro-orbital injection in subcutaneous xenograft mouse model of prostate tumor (DU145)	Inhibition of tumor growth and angiogenesis	[161]
Herpes simplex virus G47Δ	Angiostatin and bevacizumab (Avastin^®^)	IT * administration in orthotopic xenograft mouse model of human U87 glioblastoma	Combined treatment enhanced virus spread, tumor lysis, antiangiogenic activity and survival	[162]
Parvovirus H-1PV	Chemokines	IT * administration in a murine tumor model of subcutaneous implanted Kaposi sarcoma cells	Inhibition of tumor growth and VEGF expression	[163]

* IT: Intratumoral; ** IV: Intravenous.

**Table 3:** VEGF-based anti-cancer AAV vectors.

Serotype	Transgen	Assay and Experimental Model	Results	References
AAV1	Soluble VEGFR1	Skeletal muscle administration in mouse models of subcutaneous and intraperitoneal ovarian cancer	Inhibition of tumor growth and peritoneal dissemination with no adverse events	[166]
AAV2	Truncated soluble VEGFR2	Intraportal injection in orthotopic murine models of pediatric kidney tumors	Restriction of tumor development and growth	[167]
AAV2	Truncated soluble VEGFR2	Intraportal injection in murine model of metastatic neuroblastoma	Inhibition of liver metastasis and tumor vascularity with longer survival	[168]
AAV2	Soluble VEGFR1	Xenograft murine model of human ovarian tumor (SKOV3.ip1)	Tumor inhibition and increased disease-free survival	[169]
AAV2	VEGF-Trap	Single intravenous injection in mouse model of breast carcinoma	Suppression of tumor growth and metastases with reduced tumor vascularization	[170]
AAV2	VEGF-Trap + paclitaxel	Single intravenous injection in xenograft mouse model of triple negative breast cancer (TNBC)	Inhibition of tumor growth and angiogenesis. Synergistic effect in combination with paclitaxel	[171]
AAV2	VEGF-Trap + temozolomide (TMZ)	Single intravenous injection in rat model of C6 glioma	Inhibition of tumor growth and angiogenesis. Synergistic effect combined with TMZ	[172]
AAV8	Soluble VEGFR1/R2	Intracranial administration in orthotopic mouse model of glioblastoma	Reduction in overall tumor volume and longer survival	[173]
AAV8	Soluble VEGFR3-Fc	Intramuscular or intravenous administrations in xenograft metastatic murine model of human melanoma, kidney and prostate cancers	Inhibition of metastasis and lymphangiogenesis in melanoma, kidney, and prostate cancers	[174]
AAV8	sVEGFR2/R3 + paclitaxel + carboplatin	Intravenous injection in xenograft mouse model of ovarian cancer	Inhibition of tumor growth and angiogenesis in combined treatments with chemotherapy	[175]
AAVrh.10	Bevacizumab (Avastin^®^)	Intrapleural administration in mouse models of metastatic prostate carcinoma	Suppression of metastatic lung tumor growth and reduced tumor vascularization	[176]
AAVrh.10	Bevacizumab (Avastin^®^)	Single intraperitoneal injection in mouse model of ovarian cancer	Suppression of tumor growth and angiogenesis with higher survival	[177]
AAVrh.10	Bevacizumab (Avastin^®^)	Intracranial administration in xenograft mouse model of glioblastoma	Reduction of tumor volume and angiogenesis	[178]

**Table 4:** AAV vectors expressing VEGF.

Vector	Disease	Transgen	Assay	Results	References
AAV	Ischemic stroke	VEGF-A	Rat model of ischemic stroke	Increase functional angiogenesis and neurogenesis	[186]
AAV8	Hepatic encephalopathy	VEGF-C	Cirrhotic rat model	Increase lympho-angiogenesis and disease improvement	[187]
AAV9	Ischemic stroke	VEGF-C	Murine model of ischemic stroke	Increase neurogenesis and neuroprotection in pretreated animals	[188]
AAV9	Spinal cord injury	VEGF-A	Murine model of spinal cord injury	Locomotor function recovery and tissue damage protection in pretreated animals	[189]
AAV9	Segmental bone defects	VEGF-A +PDGF	Yucatan mini-pig model of tibial diaphyseal defect	Bone revascularization, remodeling and healing, but development of vascular tumors	[190]
